# Vitamin D deficiency and risk of heart failure in patients with obstructive sleep apnea: a cohort analysis

**DOI:** 10.3389/fnut.2026.1755607

**Published:** 2026-02-19

**Authors:** Ying-Jen Chang, Hsiu-Lan Weng, Kuo-Chuan Hung, Chun-Ning Ho, Yi-Chen Lai, Jheng-Yan Wu, I-Wen Chen

**Affiliations:** 1 Department of Anesthesiology, Chi Mei Medical Center, Tainan, Taiwan; 2 Department of Anesthesiology, E-Da Hospital, I-Shou University, Kaohsiung, Taiwan; 3 Department of Nutrition, Chi Mei Medical Center, Tainan, Taiwan; 4 Department of Anesthesiology, Chi Mei Medical Center, Liouying, Tainan, Taiwan

**Keywords:** cardiovascular disease, heart failure, mortality, obstructive sleep apnea, propensity score matching, vitamin D deficiency

## Abstract

**Background:**

Obstructive sleep apnea (OSA) is independently associated with increased cardiovascular morbidity, and vitamin D deficiency is highly prevalent in OSA patients. However, whether vitamin D deficiency is associated with an elevated risk of heart failure, specifically within the OSA population, remains unclear.

**Methods:**

We conducted a retrospective cohort analysis using data from the TriNetX Global Collaborative Network (2010–2022) to investigate whether vitamin D deficiency, defined as a 25-hydroxyvitamin D concentration < 20 ng/mL, is associated with new-onset heart failure in adults diagnosed with OSA. Patients with sufficient vitamin D levels (≥30 ng/mL) served as controls. Propensity score matching (1:1) was performed to balance the baseline characteristics. The primary outcome was incident heart failure; secondary outcomes included all-cause mortality, secondary pulmonary hypertension, primary pulmonary hypertension, and pulmonary embolism at the 5-year follow-up.

**Results:**

After propensity score matching, 36,497 patients were included in each cohort. Vitamin D deficiency was significantly associated with a higher risk of heart failure (HR 1.45; 95% CI 1.37–1.53; *p* < 0.001), all-cause mortality (HR 1.76; 95% CI 1.64–1.89; *p* < 0.001), secondary pulmonary hypertension (HR 1.25; 95% CI 1.13–1.38; *p* < 0.001), and pulmonary embolism (HR 1.31; 95% CI 1.17–1.47; *p* < 0.001). No significant association was observed with primary pulmonary hypertension (HR 1.22; 95% CI 0.84–1.79; *p* = 0.300). Dose-response analysis revealed attenuated associations for vitamin D insufficiency (20–29.9 ng/mL). Subgroup analyses demonstrated a stronger association among obese patients (*p* for interaction = 0.028).

**Conclusion:**

Among adults with OSA, vitamin D deficiency was linked to a markedly higher risk of developing heart failure as well as other unfavorable cardiopulmonary events, and this association demonstrated a clear dose–response pattern. These findings suggest that vitamin D status may represent an important factor in cardiovascular risk stratification in patients with OSA.

## 1 Introduction

Obstructive sleep apnea (OSA) is a highly prevalent sleep-disordered breathing condition characterized by repetitive episodes of upper airway obstruction during sleep, leading to intermittent hypoxia, sleep fragmentation, and sympathetic nervous system activation (1–3). The global burden of OSA continues to rise alongside increasing rates of obesity and aging populations, with estimates suggesting that the condition affects a substantial proportion of adults worldwide (4–6). Beyond its immediate impact on sleep quality and daytime functioning, OSA has emerged as an independent risk factor for a broad spectrum of cardiovascular complications including hypertension, coronary artery disease, arrhythmias, and heart failure (7–9). The pathophysiological mechanisms linking OSA to cardiovascular disease are multifactorial and include chronic intermittent hypoxia, oxidative stress, systemic inflammation, endothelial dysfunction, and metabolic dysregulation (7–9). These interconnected pathways contribute to adverse cardiac remodeling and may accelerate the progression toward heart failure in susceptible individuals. Given the substantial overlap between OSA and cardiovascular morbidity, identifying modifiable factors that may influence this relationship has important clinical implications for risk stratification and preventive strategies.

Vitamin D deficiency (VDD) has been implicated in the development of cardiovascular diseases through its pleiotropic effects on neurohormonal, endothelial, and inflammatory pathways (10–16). Notably, VDD is highly prevalent among patients with OSA, potentially due to shared risk factors such as obesity and metabolic dysfunction (17–19). However, whether VDD is independently associated with an elevated risk of heart failure, specifically in patients with OSA, remains unclear. Accordingly, we designed a retrospective cohort analysis based on a large federated electronic health record network to assess their relationship in adults with OSA and to further explore possible dose–response patterns and variations in risk across key clinically relevant subgroups.

## 2 Methods

### 2.1 Study design and data source

This retrospective cohort investigation was conducted using the TriNetX Research Network (TriNetX, LLC, Cambridge, MA, USA), a federated electronic health record database. The TriNetX database has been widely utilized and cited in numerous peer-reviewed observational studies across diverse clinical domains (20–22). In this study, we sought to determine whether VDD is associated with the development of new-onset heart failure in adults with OSA, thereby clarifying whether a low vitamin D status identifies a subgroup of OSA patients at particularly elevated cardiovascular risk. The research protocol was approved by the Institutional Review Board of Chi Mei Medical Center, which waived the requirement for informed consent because only de-identified data were used.

### 2.2 Study population and cohort definitions

The study population comprised adults with a previous diagnosis of OSA. OSA was identified using the International Classification of Diseases, Tenth Revision, Clinical Modification (ICD-10-CM) code G47.33, with at least one documented diagnosis recorded prior to or on the index date in the TriNetX database. Patients were classified into two cohorts based on their serum 25-hydroxyvitamin D (25 [OH] D) levels. The exposure cohort consisted of patients with vitamin D deficiency (VDD), defined as a serum 25(OH)D level of < 20 ng/mL. The control cohort comprised patients with sufficient vitamin D status, operationalized as a serum 25(OH)D concentration ≥30 ng/mL, and without any documented measurement in the deficient range during the baseline period. To ensure persistent vitamin D status throughout the observation period, patients in the deficiency cohort were required to have at least one additional measurement confirming levels below 20 ng/mL within 3 months to 5 years after the index date, with no subsequent measurements reaching 20 ng/mL or above. Similarly, individuals assigned to the control cohort, defined as levels ≥30 ng/mL, were required to consistently exhibit 25(OH)D concentrations ≥30 ng/mL with no recorded values below 30 ng/mL during the defined assessment window.

The index date for each participant was designated as the earliest vitamin D measurement that fulfilled the eligibility criteria for the respective cohort. 25(OH)D levels were obtained from laboratory records available in the TriNetX database. As TriNetX aggregates de-identified electronic health record data from multiple healthcare organizations, detailed information regarding assay methods, laboratory platforms, or calibration procedures used to measure 25(OH)D was not available. Therefore, vitamin D status in this study was defined based on recorded serum 25(OH)D values rather than specific analytical techniques. To ensure adequate follow-up, patients were required to have at least one documented healthcare visit within 3 months to 5 years of the index date.

### 2.3 Exclusion criteria

Patients were excluded if they were diagnosed with primary or secondary pulmonary hypertension at baseline or within 3 months after the index date. To minimize the influence of acute illness on vitamin D levels, patients with acute kidney failure, sepsis, COVID-19, or ICU admission were excluded. We excluded individuals with advanced chronic kidney disease because severe renal impairment profoundly alters the vitamin D metabolism. Additionally, patients with pre-existing conditions that could confound the relationship between vitamin D status and cardiovascular outcomes, including heart failure, pulmonary embolism, chronic pulmonary embolism, congenital malformations of the circulatory system, and human immunodeficiency virus infection, were excluded.

### 2.4 Propensity score matching

Baseline covariates included demographic characteristics (age, sex, and race), lifestyle factors (e.g., nicotine dependence), cardiovascular and metabolic comorbidities (e.g., ischemic heart disease and hypertension), chronic organ dysfunction (e.g., chronic kidney disease), anemia, neoplasms, and long-term steroid use. In addition, the use of continuous positive airway pressure (CPAP) for OSA and cardiometabolic medications (e.g., glucagon-like peptide-1 receptor agonists, sodium–glucose cotransporter 2 inhibitors) as well as laboratory markers (e.g., albumin, hemoglobin A1c) were incorporated into the propensity score model (Supplementary Table 1). The use of CPAP therapy was identified based on relevant prescription and procedure records within the database. Detailed information on CPAP adherence, including nightly usage duration or compliance metrics, was not available on the TriNetX platform; therefore, CPAP exposure in this study reflects documented treatment initiation rather than verified long-term adherence. Propensity score matching was performed in a 1:1 ratio between the VDD and control cohorts based on all listed characteristics. One-to-one greedy nearest-neighbor matching without replacement was performed using a caliper width of 0.1 standard deviations of the logit of the propensity score. Covariate balance between cohorts was evaluated using standardized mean differences, with values < 0.1 indicating adequate balance.

### 2.5 Outcome definition and follow-up

The primary outcome was incident heart failure, while secondary outcomes included secondary pulmonary hypertension, primary pulmonary hypertension, pulmonary embolism, and all-cause mortality at the 5-year follow-up. Outcomes were assessed during follow-up windows beginning 90 days after the index date and extending to 3 and 5 years, allowing for a latency period between exposure and outcome development while enabling the evaluation of both medium-term and long-term associations.

### 2.6 Dose-response analysis

To evaluate the potential dose-response relationships between vitamin D status and adverse outcomes, an additional analysis was conducted to compare patients with vitamin D insufficiency against the control cohort. Vitamin D insufficiency was defined as serum 25(OH)D levels between 20 and 29.9 ng/mL. This intermediate-exposure cohort was then compared with patients who consistently had sufficient vitamin D levels by applying the same matching approach and outcome evaluation strategy over a 5-year follow-up period. This study aimed to determine whether a graded association exists between declining vitamin D levels and the risk of heart failure.

### 2.7 Subgroup analysis

Patients were stratified by sex (male vs. female), age (18–50 years vs. >50 years), and the presence or absence of hypertension, obesity, cancer, hyperlipidemia, and diabetes mellitus. Within each stratum, new propensity score matching was carried out to achieve covariate balance between the vitamin D–deficient and control cohorts. Effect modification was evaluated by introducing interaction terms, and *p-values* for these interactions were reported to indicate whether the relationship between VDD and heart failure differed across subgroups.

### 2.8 Statistical analysis

Kaplan–Meier survival analysis was performed to estimate the cumulative incidence of each outcome across predefined follow-up periods, with time zero defined as the index date. Patients were censored at the time of the last available follow-up, death (for non-mortality outcomes), or the end of the observation window, whichever occurred first. Between-group differences in the survival distribution were evaluated using the log-rank test. Hazard ratios (HRs) with corresponding 95% confidence intervals (CIs) were calculated using Cox proportional hazard regression models. The proportional hazards assumption was assessed by inspecting Schoenfeld residuals and testing for time-dependent effects. All time-to-event analyses were conducted on propensity score-matched cohorts to reduce confounding by baseline imbalances. A two-sided *p-value* of less than 0.05 was considered statistically significant.

## 3 Results

### 3.1 Patient selection and baseline characteristics

**Figure 1:**
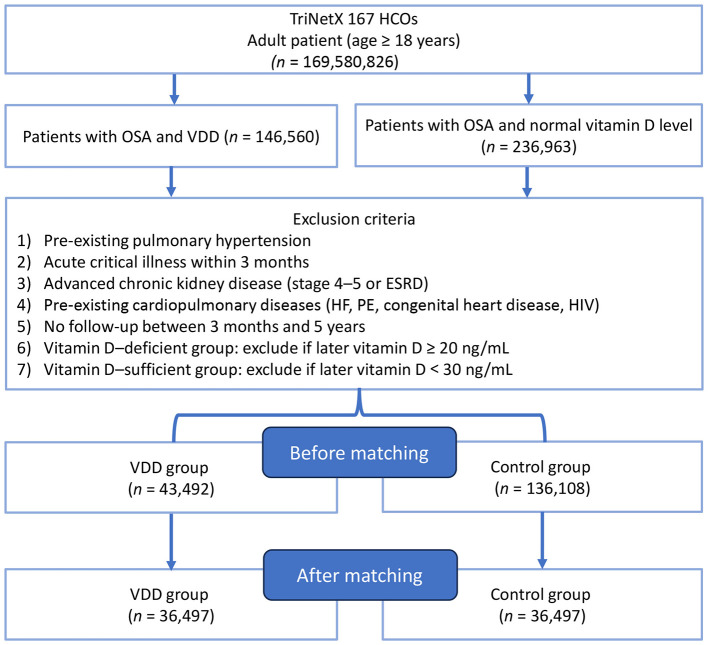
Patient selection flowchart from the TriNetX database. HCOs, healthcare organizations; OSA, obstructive sleep apnea; VDD, vitamin D deficiency; HF, heart failure; PE, pulmonary embolism; ESRD, end-stage renal disease; HIV, human immunodeficiency virus.

Using TriNetX, we identified 43,492 adults with OSA who had VDD and 136,108 adults with sufficient vitamin D levels that satisfied all predefined inclusion and exclusion criteria (Figure 1). After applying propensity score matching in a 1:1 ratio, 36,497 patients remained in each cohort for analysis. Before matching, there were notable differences between cohorts in age (46.9 ± 17.0 vs. 59.5 ± 14.3 years), racial distribution (54.6% vs. 81.6% White), and several comorbidities (e.g., hypertension, hyperlipidemia, nicotine dependence) (Table 1). Following propensity score matching, all baseline characteristics achieved excellent balance. The matched cohorts were comparable in mean age (50.1 ± 15.6 vs. 50.0 ± 15.8 years), sex distribution (52.8% vs. 52.6% female), obesity (BMI ≥ 30 kg/m^2^) prevalence (61.9% vs. 62.4%), and rates of hypertension, diabetes mellitus, chronic kidney disease, and other relevant comorbidities. Medication use and CPAP therapy were also well balanced between the groups.

**Table 1:** Baseline characteristics of patients with obstructive sleep apnea before and after propensity score matching.

**Variables**	**Before matching**			**After matching**		
	**VDD group (*****n*** = **43,492)**	**Control group (*****n*** = **136,108)**	**SMD** ^†^	**VDD group (*****n*** = **36,497)**	**Control group (*****n*** = **36,497)**	**SMD** ^†^
**Patient characteristics**						
Age at index (years)	46.9 ± 17.0	59.5 ± 14.3	0.804	50.1 ± 15.6	50.0 ± 15.8	0.002
Female	22,916 (52.7)	77,718 (57.1)	0.089	19,266 (52.8)	19,196 (52.6)	0.004
BMI ≥ 30 kg/m^2^	26,891 (61.8)	76,453 (56.2)	0.115	22,589 (61.9)	22,771 (62.4)	0.010
White	23,743 (54.6)	111,017 (81.6)	0.604	23,179 (63.5)	23,197 (63.6)	0.001
Black or African American	12,597 (29.0)	12,462 (9.2)	0.521	7,696 (21.1)	7,692 (21.1)	0.000
Asian	796 (1.8)	3,272 (2.4)	0.040	762 (2.1)	698 (1.9)	0.013
**Comorbidities**						
Overweight and obesity	26,596 (61.2)	64,870 (47.7)	0.273	21,277 (58.3)	21,356 (58.5)	0.004
Essential (primary) hypertension	24,020 (55.2)	86,600 (63.6)	0.172	21,157 (58.0)	21,203 (58.1)	0.003
Hyperlipidemia	19,284 (44.3)	84,184 (61.9)	0.356	17,743 (48.6)	17,620 (48.3)	0.007
Diabetes mellitus	12,760 (29.3)	39,355 (28.9)	0.009	11,051 (30.3)	10,986 (30.1)	0.004
Neoplasms	10,684 (24.6)	50,975 (37.5)	0.281	9,945 (27.2)	9,824 (26.9)	0.007
Nicotine dependence	7,318 (16.8)	12,455 (9.2)	0.230	5,686 (15.6)	5,675 (15.5)	0.001
Ischemic heart diseases	4,806 (11.1)	21,234 (15.6)	0.134	4,444 (12.2)	4,367 (12.0)	0.006
Diseases of liver	4,929 (11.3)	16,200 (11.9)	0.018	4,385 (12.0)	4,251 (11.6)	0.011
Other anemias	5,286 (12.2)	16,778 (12.3)	0.005	4,370 (12.0)	4,321 (11.8)	0.004
COPD	3,977 (9.1)	11,634 (8.5)	0.021	3,484 (9.5)	3,426 (9.4)	0.005
Chronic kidney disease	2,442 (5.6)	12,560 (9.2)	0.138	2,262 (6.2)	2,253 (6.2)	0.001
Alcohol related disorders	1,830 (4.2)	3,940 (2.9)	0.071	1,504 (4.1)	1,445 (4.0)	0.008
Long term use of steroids	1,400 (3.2)	4,805 (3.5)	0.017	1,203 (3.3)	1,174 (3.2)	0.004
COVID-19	949 (2.2)	3,599 (2.6)	0.030	841 (2.3)	803 (2.2)	0.007
Venous embolism and thrombosis	944 (2.2)	3,105 (2.3)	0.008	817 (2.2)	797 (2.2)	0.004
Nonrheumatic mitral valve disorders	854 (2.0)	5,513 (4.1)	0.122	815 (2.2)	878 (2.4)	0.011
Nonrheumatic aortic valve disorders	602 (1.4)	4,204 (3.1)	0.115	577 (1.6)	568 (1.6)	0.002
**Laboratory data**						
Albumin g/dL (≥3.5 g/dL)	28,674 (65.9)	95,745 (70.3)	0.095	24,638 (67.5)	24,497 (67.1)	0.008
eGFR > 60 ml/min/1.73 m^2^	31,183 (71.7)	1,02,087 (75.0)	0.075	27,069 (74.2)	26,937 (73.8)	0.008
Hemoglobin A1c >7%	6,782 (15.6)	17,802 (13.1)	0.072	5,745 (15.7)	5,669 (15.5)	0.006
Hemoglobin >12 g/dL	30,052 (69.1)	99,123 (72.8)	0.082	25,895 (71.0)	25,785 (70.7)	0.007
**Medication**						
Antilipemic agents	13,377 (30.8)	60,427 (44.4)	0.284	12,404 (34.0)	12,344 (33.8)	0.003
Insulin	7,073 (16.3)	18,053 (13.3)	0.085	5,922 (16.2)	5,914 (16.2)	0.001
Vitamin D supplement	5,958 (13.7)	35,531 (26.1)	0.315	5,637 (15.4)	5,800 (15.9)	0.012
Antianginals	3,342 (7.7)	11,331 (8.3)	0.024	2,913 (8.0)	2,869 (7.9)	0.004
GLP-1 analogs	2,225 (5.1)	7,891 (5.8)	0.030	1,997 (5.5)	2,041 (5.6)	0.005
SGLT2 inhibitors	1,092 (2.5)	4,081 (3.0)	0.030	1,005 (2.8)	1,044 (2.9)	0.006
**Treatment of OSA**						
CPAP	1,482 (3.4)	5,151 (3.8)	0.020	1,294 (3.5)	1,219 (3.3)	0.011

Data are presented as mean ± standard deviation for continuous variables and *n* (%) for categorical variables.

BMI, body mass index; COPD, chronic obstructive pulmonary disease; CPAP, continuous positive airway pressure; eGFR, estimated glomerular filtration rate; GLP-1, glucagon-like peptide-1; OSA, obstructive sleep apnea; SGLT2, sodium-glucose cotransporter-2; SMD, standardized mean difference; VDD, vitamin D deficiency.

^†^SMD values < 0.1 indicate adequate balance between groups after matching.

### 3.2 Cardiopulmonary outcomes at 5-year follow-up

During a mean follow-up of approximately 4 years (1,420 vs. 1,465 days in the VDD and control cohorts, respectively), VDD was significantly associated with higher rates of incident heart failure than the control group (7.7% vs. 5.6%; HR 1.45; 95% CI 1.37–1.53; *p* < 0.001; Table 2). All-cause mortality was also significantly higher among patients with VDD (HR 1.76; 95% CI 1.64–1.89; *p* < 0.001). With respect to other secondary outcomes, VDD demonstrated significant associations with secondary pulmonary hypertension (HR 1.25; 95% CI 1.13–1.38; *p* < 0.001) and pulmonary embolism (HR 1.31; 95% CI 1.17–1.47; *p* < 0.001). However, no statistically significant association was observed between VDD and primary pulmonary hypertension (HR 1.22; 95% CI 0.84–1.79; *p* = 0.300).

**Table 2:** Association between vitamin D deficiency and cardiopulmonary complications at 5-year follow-up.

**Outcomes**	**VDD group (*n* = 36,497)**	**Control group (*n* = 36,497)**	**HR (95% CI)**	***p*-value**
	**Events (%)**	**Events (%)**		
Heart failure	2,824 (7.7%)	2,041 (5.6%)	1.45 (1.37–1.53)	< 0.001
Mortality	2,119 (5.8%)	1,247 (3.4%)	1.76 (1.64–1.89)	< 0.001
Secondary pulmonary hypertension	899 (2.5%)	745 (2.0%)	1.25 (1.13–1.38)	< 0.001
Primary pulmonary hypertension	58 (0.16%)	49 (0.13%)	1.22 (0.84–1.79)	0.300
Pulmonary embolism	647 (1.8%)	510 (1.4%)	1.31 (1.17–1.47)	< 0.001

Values are expressed as event counts (percentages) unless otherwise indicated.

VDD, vitamin D deficiency; HR, hazard ratio; CI, confidence interval.

### 3.3 Cardiopulmonary outcomes at 3-year follow-up

At the 3-year follow-up, the overall pattern of results remained unchanged (Table 3). VDD continued to be associated with higher incidences of heart failure (HR 1.50; 95% CI 1.40–1.61; *p* < 0.001) and all-cause mortality (HR 1.89; 95% CI 1.73–2.06; *p* < 0.001), as shown in Table 3. Elevated risks were also noted for secondary pulmonary hypertension (HR 1.22; 95% CI 1.09–1.38; *p* < 0.001) and pulmonary embolism (HR 1.41; 95% CI 1.23–1.61; *p* < 0.001), both remaining statistically significant at this time point. Primary pulmonary hypertension did not show a significant association with VDD at three-year follow-up (HR 1.15; 95% CI 0.74–1.79; *p* = 0.541).

**Table 3:** Association between vitamin D deficiency and cardiopulmonary complications at 3-year follow-up.

**Outcomes**	**VDD group (*n* = 36,497)**	**Control group (*n* = 36,497)**	**HR (95% CI)**	***p*-value**
	**Events (%)**	**Events (%)**		
Heart failure	1,974 (5.4%)	1,376 (3.8%)	1.50 (1.40–1.61)	< 0.001
Mortality	1,501 (4.1%)	826 (2.3%)	1.89 (1.73–2.06)	< 0.001
Secondary pulmonary hypertension	588 (1.6%)	500 (1.4%)	1.22 (1.09–1.38)	< 0.001
Primary pulmonary hypertension	41 (0.11%)	37 (0.10%)	1.15 (0.74–1.79)	0.541
Pulmonary embolism	480 (1.3%)	354 (1.0%)	1.41 (1.23–1.61)	< 0.001

Values are expressed as event counts (percentages) unless otherwise indicated.

VDD, vitamin D deficiency; HR, hazard ratio; CI, confidence interval.

### 3.4 Dose-response analysis

To evaluate potential dose-response relationships, we compared patients with vitamin D insufficiency (25(OH)D 20–29.9 ng/mL) against those with sufficient vitamin D levels (Table 4). After propensity score matching (*n* = 51,582 per group), vitamin D insufficiency was associated with modestly elevated risks of heart failure (HR 1.15; 95% CI 1.09–1.21; *p* < 0.001), all-cause mortality (HR 1.36; 95% CI 1.28–1.44; *p* < 0.001), and pulmonary embolism (HR 1.15; 95% CI 1.04–1.27; *p* = 0.008) at five-year follow-up. Notably, the HRs in the insufficiency group were lower than those in the deficiency group, indicating a stepwise increase in cardiopulmonary risk as the vitamin D concentration declined.

**Table 4:** Association between vitamin D insufficiency and cardiopulmonary complications at 5-year follow-up.

**Outcomes**	**VDI group (*n* = 51,582)**	**Control group (*n* = 51,582)**	**HR (95% CI)**	***p-value***
	**Events (%)**	**Events (%)**		
Heart failure	3,336 (6.5%)	2,995 (5.8%)	1.15 (1.09–1.21)	< 0.001
Mortality	2,425 (4.7%)	1,839 (3.6%)	1.36 (1.28–1.44)	< 0.001
Secondary pulmonary hypertension	1,054 (2.0%)	1,043 (2.0%)	1.04 (0.95–1.13)	0.379
Primary pulmonary hypertension	69 (0.13%)	61 (0.12%)	1.17 (0.83–1.64)	0.385
Pulmonary embolism	780 (1.5%)	698 (1.4%)	1.15 (1.04–1.27)	0.008

Values are expressed as event counts (percentages) unless otherwise indicated.

VDI, vitamin D insufficiency; HR, hazard ratio; CI, confidence interval.

### 3.5 Subgroup analysis

**Figure 2:**
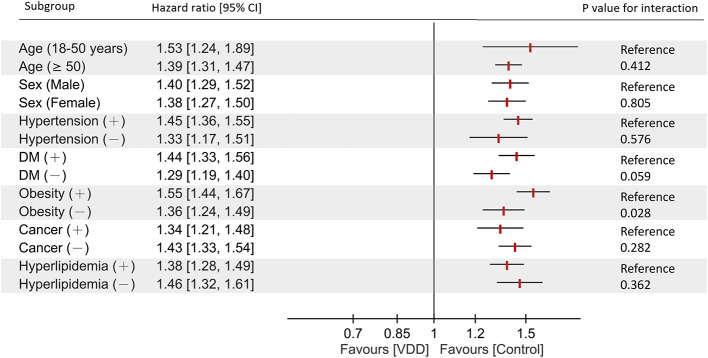
Forest plot of subgroup analyses examining the association between vitamin D deficiency and incident heart failure at 5-year follow-up. Hazard ratios with 95% confidence intervals are shown for each subgroup after propensity score matching. *P-values* for interaction assess effect modification across subgroups. A significant interaction was observed for obesity (*p* = 0.028). CI, confidence interval; DM, diabetes mellitus; VDD, vitamin D deficiency.

In a priori–defined subgroup analyses, the relationship between VDD and new-onset heart failure over the 5-year observation period was generally consistent across the various clinical strata (Figure 2). The association remained significant regardless of sex, age category, and the presence or absence of hypertension, cancer, hyperlipidemia, or diabetes mellitus, with no significant interaction effects observed for these variables. However, a significant interaction was identified for obesity status (*p* for interaction = 0.028), with obese patients showing a stronger association between VDD and heart failure (HR 1.55; 95% CI 1.44–1.67) than non-obese patients (HR 1.36; 95% CI 1.24–1.49).

## 4 Discussion

The present study investigated the association between VDD and incident heart failure among adults with OSA using a large federated health research network. In this cohort, individuals with VDD had a roughly 45% greater risk of developing heart failure during the 5-year follow-up than those with sufficient vitamin D levels. This association persisted after rigorous propensity score matching and was consistent across both 3-year and 5-year follow-up windows. Furthermore, we observed a graded relationship between vitamin D status and heart failure risk, with patients exhibiting vitamin D insufficiency demonstrating an intermediate-risk elevation (HR 1.15) compared to those with VDD (HR 1.45). Subgroup analyses revealed consistent associations across most clinical subgroups, with a notably stronger association observed among obese patients.

Our findings align with and extend prior epidemiological evidence linking VDD to cardiovascular outcomes (15, 16). In the Framingham Offspring Study, individuals with 25(OH)D levels below 15 ng/mL exhibited a 62% increased risk of cardiovascular events during 5.4 years of follow-up, with the association being particularly pronounced among hypertensive individuals (23). A meta-analysis of seven cohort studies encompassing 5,941 patients with heart failure reported that low vitamin D status was associated with a 37% increased risk of all-cause mortality (24). Our observed HR of 1.45 for incident heart failure falls within the range of effect sizes reported in these investigations, suggesting biological consistency across different populations. Notably, a recent bidirectional Mendelian randomization study provided genetic evidence supporting a causal relationship between vitamin D status and heart failure risk, thus strengthening the plausibility of our observational findings (25).

Critically, prior studies were conducted predominantly in the general population or among patients with established heart failure, rather than specifically examining OSA patients at risk of developing heart failure. Although meta-analyses have consistently demonstrated that OSA patients have significantly lower serum 25(OH)D levels than controls regardless of age or BMI, and that this deficiency worsens with increasing OSA severity (18, 19), no prior study has directly examined whether VDD confers additional heart failure risk specifically within the OSA population. Our investigation revealed that among patients with OSA, VDD is associated with an elevated risk of heart failure. Together, these studies suggest a bidirectional relationship between vitamin D status and sleep-disordered breathing, which may have cumulative cardiovascular consequences.

Several biological mechanisms may underlie this association. Vitamin D modulates the renin-angiotensin-aldosterone system by suppressing renin gene expression, and deficiency may promote hypertension, myocardial hypertrophy, and fibrosis through inappropriate neurohormonal activation (26, 27). Vitamin D also possesses anti-inflammatory properties, regulating pro-inflammatory cytokines (28, 29). Given that OSA is characterized by chronic low-grade systemic inflammation driven by intermittent hypoxia, superimposed VDD may amplify inflammatory cascades, contributing to endothelial dysfunction and adverse cardiac remodeling. Additionally, vitamin D influences calcium homeostasis in cardiomyocytes and has been linked to insulin resistance (30), a condition prevalent among OSA patients that independently contributes to heart failure risk (31, 32). The convergence of these pathways in patients with OSA and concomitant VDD may create a particularly adverse milieu for cardiac health.

The dose-response relationship observed in our study provides additional support for biological plausibility. The attenuated HR among patients with vitamin D insufficiency (HR 1.15) compared to those with VDD (HR 1.45) suggests that the relationship follows a continuous gradient rather than a simple threshold effect. Such dose-response patterns strengthen epidemiological associations by reducing the likelihood of confounding and aligning with causal frameworks. Nevertheless, as an observational study, causality cannot be established and residual confounding by unmeasured factors cannot be entirely excluded.

The significant association between VDD and obesity merits further investigation. Our subgroup analyses revealed a stronger association among obese patients (HR 1.55) than among non-obese individuals (HR 1.36). This finding aligns with evidence that adipose tissue sequesters vitamin D, reducing its bioavailability despite comparable total serum concentrations (33, 34). Similarly, the Jackson Heart Study observed that higher 25(OH)D levels were more strongly associated with reduced heart failure risk and inhibited left ventricular concentric remodeling in certain subgroups (35). Therefore, the combination of OSA, obesity, and VDD may constitute a particularly high-risk phenotype that deserves targeted clinical attention, as these three conditions share overlapping pathophysiological pathways involving inflammation, metabolic dysfunction, and neurohormonal activation.

Our study had several methodological strengths. The large sample size derived from 167 healthcare organizations provided adequate statistical power and enhanced generalizability. Confirmatory vitamin D measurements ensured that exposure classification reflected sustained vitamin D status rather than transient fluctuations. Extensive propensity score matching achieved excellent covariate balance, with all standardized mean differences below 0.1. The exclusion of patients with baseline heart failure, advanced chronic kidney disease, and recent acute illnesses minimized reverse causation and confounding factors substantially affecting vitamin D metabolism.

This study has several limitations that warrant consideration. First, its retrospective observational design precludes the establishment of causality between VDD and heart failure. Despite rigorous propensity score matching, residual confounding from unmeasured factors (e.g., diet, sunlight exposure, parathyroid hormone levels, and socioeconomic status) and potential reverse causation cannot be fully excluded, as conditions and behaviors (e.g., obesity, reduced physical activity, chronic illness burden, or systemic inflammation) associated with a higher risk of heart failure may contribute to lower circulating vitamin D levels. Second, the TriNetX database relies on diagnostic codes entered during routine clinical care, which may introduce misclassification bias for both exposure and outcome ascertainment. Third, although we required confirmatory vitamin D measurements to ensure a sustained exposure status, we could not account for vitamin D supplementation adherence or dosing between measurements, potentially attenuating the observed associations. In addition, reliance on single or limited laboratory assessments may not fully capture long-term vitamin D status over the follow-up period. Fourth, objective measures of OSA severity, including the apnea–hypopnea index and oxygen desaturation parameters, were not available in the TriNetX database, precluding assessment of effect modification or dose–response relationships according to disease severity. Fifth, CPAP adherence could not be comprehensively assessed beyond prescription records, and treatment compliance may have substantially influenced the cardiovascular outcomes. Sixth, we lacked information regarding heart failure subtypes, precluding differentiation between heart failure with preserved and reduced ejection fractions. Finally, while the multi-institutional nature of TriNetX enhances generalizability, the predominance of healthcare organizations from specific geographic regions may limit its applicability to other populations.

## 5 Conclusion

This large retrospective cohort study demonstrates that VDD is associated with a significantly increased risk of incident heart failure among adults with OSA, with evidence of a dose-response relationship. These findings address a previously unexamined knowledge gap and underscore the potential importance of vitamin D status in the cardiovascular health of patients with OSA. Given the absence of objective measures of obstructive sleep apnea severity, such as the apnea–hypopnea index, our findings should be interpreted with appropriate caution and viewed as hypothesis-generating rather than confirmatory. Prospective interventional studies are required to determine whether correction of VDD can modify heart failure risk before any clinical recommendations can be made.

## Data Availability

The raw data supporting the conclusions of this article will be made available by the authors, without undue reservation.
