# Supplementary material for: Vitamin D deficiency and risk of heart failure in patients with obstructive sleep apnea: a cohort analysis
Source: Front Nutr. 2026 Feb 19;13:1755607. doi: 10.3389/fnut.2026.1755607 (PMC12960163; doi:10.3389/fnut.2026.1755607)
Supplement: Supplementary file 1 [file Table_1.docx]

**Supplemental table 1. Code for variables and outcomes**

| **Variables** | **Code system** | **Code** |
| --- | --- | --- |
| Nicotine dependence | ICD-10-CM | F17 |
| Alcohol related disorders | ICD-10-CM | F10 |
| Chronic kidney disease (CKD) | ICD-10-CM | N18 |
| Diseases of liver | ICD-10-CM | K70–K77 |
| Neoplasms | ICD-10-CM | C00–D49 |
| Long term (current) use of steroids | ICD-10-CM | Z79.5 |
| COVID-19 | ICD-10-CM | U07.1 |
| Ischemic heart diseases | ICD-10-CM | I20–I25 |
| Disorders of lipoprotein metabolism and other lipidemias | ICD-10-CM | E78 |
| Other venous embolism and thrombosis | ICD-10-CM | I82 |
| Diabetes mellitus | ICD-10-CM | E08–E13 |
| Other chronic obstructive pulmonary disease | ICD-10-CM | J44 |
| Overweight and obesity | ICD-10-CM | E66 |
| Other anemias | ICD-10-CM | D64 |
| Nonrheumatic mitral valve disorders | ICD-10-CM | I34 |
| Nonrheumatic aortic valve disorders | ICD-10-CM | I35 |
| Heart failure（baseline 變項） | ICD-10-CM | I50 |
| Essential (primary) hypertension | ICD-10-CM | I10 |
| Continuous positive airway pressure ventilation, initiation and management | CPT | 94660 |
| Glucagon-like peptide-1 (GLP-1) analogues | ATC | A10BJ |
| Insulin | TriNetX drug | HS501 |
| Sodium-glucose co-transporter 2 (SGLT2) inhibitors | ATC | A10BK |
| Antianginals | TriNetX drug class | CV250 |
| Antilipemic agents | TriNetX drug class | CV350 |
| Vitamin D | TriNetX drug class | VT500 |
| Albumin [Mass/volume] in Serum, Plasma or Blood | TriNetX lab | 9045 |
| Hemoglobin A1c/Hemoglobin.total in Blood | TriNetX lab | 9037 |
| eGFR | TriNetX lab | 8001 |
| BMI | TriNetX lab | 9083 |
| Hemoglobin [Mass/volume] in Blood | TriNetX lab | 9014 |
